# Supplementary material for: Hidden diversity of Trypanosomatidae (Protozoa: Kinetoplastea) in bats from an urban park in Brazil
Source: PLoS One. 2025 Mar 17;20(3):e0319781. doi: 10.1371/journal.pone.0319781 (PMC11913285; doi:10.1371/journal.pone.0319781)
Supplement: Attachment — Submitted filename: Response_to_editor_and_reviewers_auresp_2.docx [file pone.0319781.s003.docx]

February 6, 2025

**Manuscript ID: PONE-D-24-46291**

**Hidden diversity of Trypanosomatidae (Protozoa: Kinetoplastea) in bats from an urban park in Brazil**

Dear Dr. Belo,

Thank you for your attention in reviewing the manuscript.

We have conducted an extensive review of the article, addressing typographical errors and misspellings as suggested by Reviewer 2. Moreover, we have added more references regarding triatomine-bat interactions, as requested.

Now, since all questions raised by the reviewers have been resolved, we are confident that the manuscript meets all the requirements for publication. We would appreciate your prompt guidance in this process.

Sincerely,

**Felipe Dutra Rêgo**

Instituto René Rachou - Oswaldo Cruz Foundation

**Review Comments to the Author**

**Reviewer #2:**

*Accept with Minor Revisions. The manuscript is well-prepared, scientifically robust, and contributes meaningful data. Minor language adjustments and additional references will further strengthen the paper.*

**Authors’ comments:** We conducted a thorough review of the manuscript, addressing typographical errors and misspellings. Additionally, we have incorporated additional references related to triatomine-bat interactions, as requested.
